# Understanding and Explaining How Staff Promote Quality for Older People Living in Long-Term Care Facilities

**DOI:** 10.1093/geroni/igab046.557

**Published:** 2021-12-17

**Authors:** Julienne Meyer, Kirsty Haunch, Carl Thompson, Karen Spilsbury

**Affiliations:** 1 City, University of London, Guildford, England, United Kingdom; 2 University of Leeds, University of Leeds, Leeds, England, United Kingdom

## Abstract

Little is known about how the workforce influences quality in long term care facilities for older people. Conceptually, quality is complex, often contested, and dynamic, has overlapping physical, social, psychological and emotional dimensions and can refer to both quality of life and quality of care. Assuming ‘more staff equates to better quality’ is intuitively appealing but research suggests that a more nuanced, non-linear, relationship exists. A programme of research in the UK is developing theoretical and empirical explanations of how staff promote quality for older people living in long-term care facilities. It shifts the debate from numbers of staff and their relationship to quality indicators toward recognising the ways in which staff more broadly influence quality. Our work will be useful for people and organisations making policy and delivering services on the best ways to deploy and support quality in long term care through the most valuable resource: its staff.

